# Supplementary material for: Low-Level Clostridial Spores’ Milk to Limit the Onset of Late Blowing Defect in Lysozyme-Free, Grana-Type Cheese
Source: Foods. 2023 May 2;12(9):1880. doi: 10.3390/foods12091880 (PMC10177814; doi:10.3390/foods12091880)
Supplement: Supplementary file 1 [file foods-12-01880-s001.zip › Figure S1.pdf]

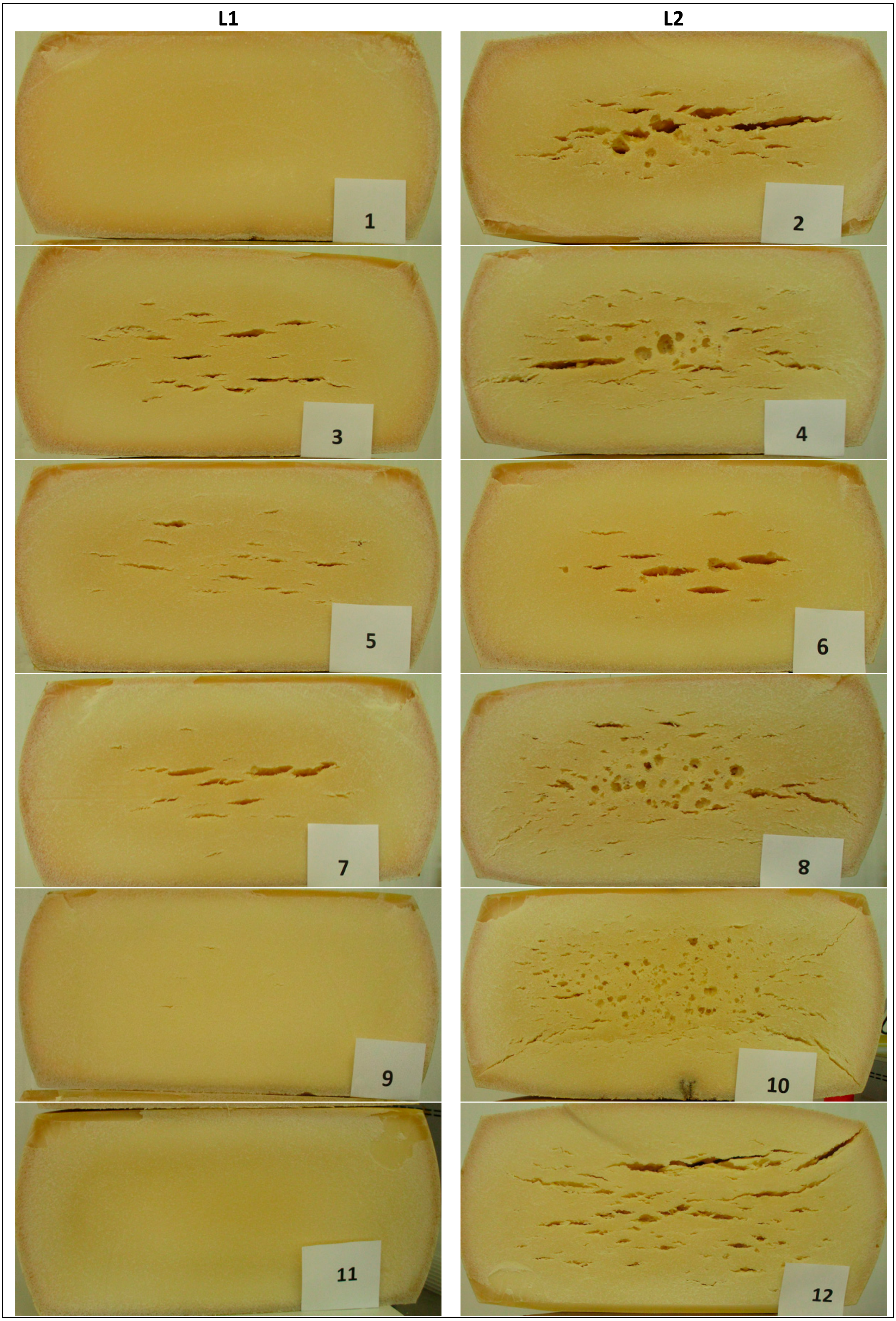
L1
L2
1
2
3
4
5
6
7
8
9
10
11
12

Figure S1. Grana-type cheeses manufactured without the use of lysozyme. Cheeses on the left column were obtained from L1 milk (BCS load < 2.0 log MPN L^{-1}); cheeses 3, 5, 7 and 1, 9, 11 were produced in the warm and cold season, respectively. Cheeses on the right column were obtained from L2 milk (BCS load ≥ 3.0 log MPN L^{-1}); cheeses 4, 6, 8 and 2, 10, 12 were produced in the warm and cold season, respectively.
