# Supplementary material for: Low-Level Clostridial Spores’ Milk to Limit the Onset of Late Blowing Defect in Lysozyme-Free, Grana-Type Cheese
Source: Foods. 2023 May 2;12(9):1880. doi: 10.3390/foods12091880 (PMC10177814; doi:10.3390/foods12091880)
Supplement: Supplementary file 1 [file foods-12-01880-s001.zip › Table S1.pdf]

Table S1. Microbiological parameters of vat milk and Grana-type cheeses.

**Cheesemaking trials**			**Vat milk**		**Cheese**				
Milk quality[1]	Climatic period[2]	trial nr	ANSB	BCS	ANSB	BCS	TMB	PB	ASB
			log MPN L^{-1}		log MPN g^{-1}		log cfu g^{-1}		
L1	C	1	3.18	3.18	-0.32	-0.32	4.79	0.70	2.65
L1	W	3	4.66	1.68	1.63	-0.32	6.04	0.70	2.74
L1	W	5	2.56	1.68	0.56	0.56	4.53	0.70	2.81
L1	W	7	3.63	1.68	0.96	0.96	4.77	0.70	2.54
L1	C	9	3.63	1.68	0.96	0.96	6.05	0.70	2.30
L1	C	11	3.36	1.68	0.56	-0.32	5.12	0.70	1.00
		average	3.50^{A}	1.93^{A}	0.73^{A}	0.25^{A}	5.21^{A}	0.70^{A}	2.34^{A}
		SD	0.69	0.61	0.65	0.65	0.67	0.00	0.68
L2	C	2	3.63	3.36	1.36	1.36	5.69	0.70	2.54
L2	W	4	2.96	1.68	4.66	4.66	6.09	0.70	3.08
L2	W	6	3.63	3.36	2.38	2.38	5.00	0.70	2.81
L2	W	8	2.96	2.96	5.04	5.04	5.51	2.74	2.60
L2	C	10	3.63	3.63	4.66	4.38	6.18	0.70	2.60
L2	C	12	3.18	2.96	3.38	3.38	6.48	0.70	1.70
		average	3.33^{A}	2.99^{B}	3.58^{B}	3.53^{B}	5.83^{A}	1.04^{A}	2.56^{A}
		SD	0.34	0.69	1.48	1.44	0.53	0.83	0.46

ANSB: anaerobic sporeforming bacteria; BCS: butyric clostridia spores; TMB: total mesophilic bacteria; PB: propionibacteria; ASB: aerobic sporeforming bacteria.

[1]milk from farms recognized for low (L1) or high (L2) content of butyric clostridia spores.

[2]climate of the period when the cheesemaking was carried out: warm (W), cool (C).

^{A,B} average values in column with different capital letters are different ($P < 0.05$)
