# Supplementary material for: Low-Level Clostridial Spores’ Milk to Limit the Onset of Late Blowing Defect in Lysozyme-Free, Grana-Type Cheese
Source: Foods. 2023 May 2;12(9):1880. doi: 10.3390/foods12091880 (PMC10177814; doi:10.3390/foods12091880)
Supplement: Supplementary file 1 [file foods-12-01880-s001.zip › Table S2.pdf]

Table S2. Milk creaming. Processing parameters and chemical composition of starting bulk milk and residual final cream.

**Cheesemaking trials**			**Bulk milk (*composition*)**				**Milk creaming process *starting parameters***			
Milk quality[1]	Climatic period[2]	trial nr	fat (%)	protein (%)	fat to protein (ratio)	dry matter (%)	environment temperature (°C)	milk temperature (°C)	milk pH	milk titratable acidity (°SH/50 ml)
L1	C	1a	4.00	3.32	1.21	13.33	17.3	10,0	6.68	3.3
		1b	3.93	3.31	1.19	13.18	17.3	10,0	6.68	3.3
L1	W	3a	3.88	3.29	1.18	13.13	20.6	9.4	6.63	3.1
		3b	3.90	3.29	1.19	13.16	20.6	8.4	6.63	3.7
L1	W	5a	3.86	3.26	1.18	13.11	22.4	10.2	6.63	3.2
		5b	3.84	3.26	1.18	13.08	22.4	10,0	6.63	3.2
L1	W	7a	3.83	3.24	1.19	13.05	23.6	10.5	6.68	3.2
		7b	3.83	3.23	1.19	13.05	23.6	10.2	6.66	3.2
L1	C	9a	4.11	3.35	1.23	13.41	21,0	10.9	6.72	3.3
		9b	4.08	3.33	1.23	13.38	21,0	10.9	6.73	3.3
L1	C	11a	3.98	3.45	1.15	13.39	20,0	10.4	6.71	3.3
		11b	3.96	3.46	1.14	13.38	20,0	10.6	6.72	3.3
		average	3.93^{A}	3.32^{A}	1.19^{A}	13.22^{A}	20.8^{A}	10.1^{A}	6.68^{A}	3.3^{A}
		SD	0.09	0.07	0.03	0.14	2.1	0.7	0.04	0.1
L2	C	2a	3.85	3.33	1.16	13.12	21.0	10.0	6.66	3.1
		2b	3.85	3.32	1.16	13.07	21.0	10.0	6.67	3.1
L2	W	4a	3.79	3.37	1.12	13.10	21.7	10.2	6.70	3.1
		4b	3.79	3.36	1.13	13.09	21.7	9.7	6.69	3.1
L2	W	6a	3.84	3.32	1.16	13.06	23.9	10.2	6.78	3.1
		6b	3.82	3.33	1.15	13.04	23.9	10.2	6.70	3.1
L2	W	8a	3.70	3.35	1.10	12.99	22.5	10.7	6.68	3.1
		8b	3.72	3.35	1.11	13.01	22.5	10.3	6.67	3.1
L2	C	10a	3.92	3.60	1.09	13.42	20.3	10.1	6.80	3.3
		10b	3.86	3.60	1.07	13.38	20.3	10.3	6.78	3.3
L2	C	12a	3.98	3.60	1.11	13.47	23.0	11.0	6.76	3.4
		12b	3.97	3.59	1.11	13.47	23.0	10.7	6.76	3.4
		average	3.84^{B}	3.43^{B}	1.12^{B}	13.19^{A}	22.1^{A}	10.3^{A}	6.72^{B}	3.2^{A}
		SD	0.09	0.13	0.03	0.19	1.3	0.4	0.05	0.1

Continue

Cheesemaking trials			Milk creaming process *ending parameters*						Cream (*composition*)		
Milk quality[1]	Climatic period[2]	trial nr	environment temperature (°C)	milk temperature (°C)	milk pH	milk titratable acidity (°SH/50 ml)	creaming time (min)	separated cream, quantity (kg)	fat (%)	protein (%)	dry matter (%)
L1	C	1a	17.4	13.0	6.61	3.4	371	28.61	21.02	2.80	nd
		1b	17.4	12.2	6.60	3.4	352	28.79	19.98	2.85	nd
L1	H	3a	21.5	13.8	6.66	3.2	334	32.23	19.49	2.84	27.26
		3b	22.2	14.0	6.65	3.2	353	31.46	19.73	2.83	27.48
L1	H	5a	23.7	15.4	6.63	3.3	360	33.77	21.67	2.76	29.31
		5b	24.1	16.0	6.73	3.2	385	34.09	22.16	2.74	29.78
L1	H	7a	24.4	16.2	6.68	3.2	360	36.52	20.93	2.76	28.57
		7b	24.7	16.1	6.66	3.3	380	38.15	21.58	2.74	29.20
L1	C	9a	21.8	15.4	6.79	3.3	406	31.31	22.57	2.81	30.29
		9b	21.8	15.3	6.79	3.3	390	31.60	21.33	2.82	29.07
L1	C	11a	20.0	15.1	6.73	3.4	418	37.40	20.96	2.89	28.80
		11b	20.0	15.1	6.73	3.4	425	35.45	21.54	2.87	29.30
		average	21.6^{A}	14.8^{A}	6.69^{A}	3.3^{A}	378^{A}	33.28^{A}	21.08^{A}	2.81^{A}	28.91^{A}
		SD	2.5	1.3	0.06	0.1	28	3.16	0.95	0.05	0.94
L2	C	2a	21.2	14.4	6.75	3.3	390	36.50	20.05	2.84	27.80
		2b	20.7	14.2	6.73	3.3	360	35.28	20.70	2.81	28.40
L2	H	4a	22.4	14.8	6.68	3.1	360	38.07	19.99	2.86	27.75
		4b	nd	14.4	6.68	3.1	370	36.05	19.81	2.87	27.59
L2	H	6a	24.2	16.1	6.67	3.2	360	39.67	20.83	2.79	28.51
		6b	nd	16.7	6.70	3.2	367	40.67	20.15	2.82	27.87
L2	H	8a	23.0	16.5	6.64	3.2	383	42.81	20.01	2.85	27.77
		8b	23.0	15.2	6.68	3.2	358	45.14	18.95	2.89	26.76
L2	C	10a	20.3	14.6	6.75	3.4	425	39.78	19.95	3.04	nd
		10b	20.3	15.0	6.76	3.4	428	39.32	19.92	3.05	nd
L2	C	12a	22.6	17.6	6.75	3.5	432	43.46	20.61	2.99	28.55
		12b	22.6	17.6	6.77	3.5	420	39.15	21.89	2.95	29.77
		average	22.0^{A}	15.6^{A}	6.71^{A}	3.3^{A}	388^{A}	39.66^{B}	20.24^{B}	2.90^{B}	28.08^{B}
		SD	1.3	1.3	0.04	0.1	30	3.02	0.71	0.09	0.79

[1]milk from farms recognized for low (L1) or high (L2) content of butyric clostridia spores.

[2]climate of the period when the cheesemaking was carried out: warm (W), cool (C)

^{A,B} average values in column with different capital letters are different (P < 0.05)
