# Supplementary material for: Low-Level Clostridial Spores’ Milk to Limit the Onset of Late Blowing Defect in Lysozyme-Free, Grana-Type Cheese
Source: Foods. 2023 May 2;12(9):1880. doi: 10.3390/foods12091880 (PMC10177814; doi:10.3390/foods12091880)
Supplement: Supplementary file 1 [file foods-12-01880-s001.zip › Table S3.pdf]

Table S3. Technical parameters recorded during cheese manufacturing trials.

**Cheesemaking trials**			**Cheesemaking parameters**										
			**Vat milk**							**Whey starter culture (WS)**			
Milk quality[1]	Climatic period[2]	trial nr	temperature (°C)	pH	titratable acidity (°SH/50 ml)	fat (%)	protein (%)	fat to casein (ratio)	dry matter (%)	pH	titratable acidity (°SH/50 ml)	temperature (°C)	quantity (g)
L1	C	1a	16.0	6.62	3.4	2.97	3.35	1.14	12.34	3.54	27.8	20.2	1300
		1b	17.2	6.58	3.4	2.97	3.35	1.14	12.30	3.54	27.8	20.2	1300
L1	W	3a	13.8	6.67	3.3	2.91	3.32	1.12	12.28	3.29	27.6	22.5	1500
		3b	13.9	6.67	3.3	2.91	3.32	1.12	12.27	3.29	27.6	22.5	1500
L1	W	5a	13.9	6.62	3.3	2.69	3.30	1.05	12.07	3.32	28.0	22.1	1600
		5b	14.1	6.65	3.3	2.71	3.30	1.05	12.09	3.32	28.0	22.1	1600
L1	W	7a	14.2	6.64	3.3	2.56	3.28	1.00	11.90	3.25	28.5	23.2	1400
		7b	14.1	6.68	3.3	2.50	3.24	0.99	11.79	3.25	28.5	23.2	1400
L1	C	9a	14.0	6.74	3.5	2.95	3.37	1.12	12.39	3.36	28.7	20.7	1500
		9b	14.0	6.74	3.5	2.95	3.37	1.12	12.40	3.36	28.7	20.7	1500
L1	C	11a	13.8	6.72	3.4	2.78	3.50	1.02	12.33	3.34	29.5	20.9	1700
		11b	13.8	6.72	3.4	2.78	3.50	1.02	12.33	3.34	29.5	20.9	1700
		average	14.4^{A}	6.67^{A}	3.4^{A}	2.81^{A}	3.35^{A}	1.07^{A}	12.21^{A}	3.35^{A}	28.4^{A}	21.6^{A}	1500^{A}
		SD	1.1	0.05	0.1	0.16	0.08	0.06	0.20	0.10	0.7	1.1	135
L2	C	2a	14.0	6.66	3.2	2.67	3.33	1.00	11.92	3.29	28.6	22.0	1500
		2b	14.2	6.63	3.2	2.67	3.33	1.00	nd	3.29	28.6	22.0	1500
L2	W	4a	14.0	6.69	3.1	2.60	3.38	0.99	12.02	3.45	28.7	22.8	1560
		4b	14.4	6.66	3.1	2.60	3.39	0.98	12.02	3.45	28.7	22.8	1560
L2	W	6a	14.1	6.64	3.1	2.53	3.37	0.96	11.90	3.29	30.0	23.0	1450
		6b	14.0	6.67	3.2	2.52	3.36	0.96	11.88	3.29	30.0	23.0	1450
L2	W	8a	13.8	6.65	3.2	2.35	3.40	0.89	11.79	3.37	29.3	24.6	1500
		8b	14.2	6.67	3.2	2.35	3.40	0.89	11.80	3.37	29.3	24.6	1580
L2	C	10a	13.8	6.76	3.4	2.61	3.65	0.92	12.27	3.36	28.4	20.7	1800
		10b	13.8	6.76	3.4	2.61	3.65	0.92	12.28	3.36	28.4	20.7	1800
L2	C	12a	14.2	6.73	3.5	2.58	3.64	0.91	12.24	3.41	27.2	20.6	1900
		12b	13.9	6.77	3.5	2.57	3.59	0.89	12.23	3.41	27.2	20.6	1900
		average	14.0^{A}	6.69^{A}	3.3^{B}	2.56^{B}	3.46^{B}	0.94^{B}	12.03^{B}	3.36^{A}	28.7^{A}	22.3^{A}	1625^{A}
		SD	0.2	0.05	0.2	0.11	0.13	0.04	0.19	0.06	0.9	1.5	173

Continue

Cheesemaking trials			Cheesemaking parameters								
			Vat milk after WS addition			Vat milk at time of rennet addition			Renneting		
Milk quality[1]	Climatic period[2]	trial nr	pH	titratable acidity (°SH/50 ml)	temperature (°C)	pH	titratable acidity (°SH/50 ml)	temperature (°C)	coagulation time (min)	manual cutting time (sec)	mechanical cutting time(sec)
L1	C	1a	6.45	4.0	18.0	6.40	4.2	33.5	12.3	39	61
		1b	6.43	4.0	18.0	6.41	4.2	33.4	12.4	46	58
L1	W	3a	6.41	4.0	22.5	6.41	4.0	33.2	11.3	60	55
		3b	6.43	3.9	21.8	6.41	3.9	33.2	11.2	37	36
L1	W	5a	6.43	4.0	21.0	6.41	4.1	33.2	10.5	56	67
		5b	6.43	4.0	21.0	6.41	4.2	33.3	10.5	105	64
L1	W	7a	6.43	4.1	21.7	6.40	4.1	33.3	11.4	44	74
		7b	6.41	4.1	21.4	6.41	4.1	33.3	11.1	42	78
L1	C	9a	6.43	4.3	21.8	6.39	4.3	33.4	9.5	38	97
		9b	6.43	4.4	21.0	6.40	4.4	33.4	9.4	55	79
L1	C	11a	6.44	4.3	21.3	6.41	4.3	33.2	10.4	39	76
		11b	6.44	4.3	21.7	6.40	4.3	33.4	9.4	30	87
		average	6.43^{A}	4.1^{A}	20.9^{A}	6.41^{A}	4.2^{A}	33.3^{A}	10.8^{A}	49^{A}	69^{A}
		SD	0.01	0.2	1.4	0.01	0.2	0.1	1.0	20	16
L2	C	2a	6.44	4.0	22.2	6.40	4.0	33.0	11.5	42	59
		2b	6.43	4.0	21.8	6.41	4.0	33.0	11.3	33	55
L2	W	4a	6.44	4.0	21.4	6.41	4.0	33.1	11.2	42	91
		4b	6.42	4.0	22.8	6.41	4.0	33.3	11.1	35	80
L2	W	6a	6.42	3.9	21.0	6.42	3.9	33.3	11.3	43	83
		6b	6.43	3.9	21.3	6.41	4.0	33.4	11.2	41	84
L2	W	8a	6.39	4.0	21.1	6.40	4.0	33.3	10.6	75	80
		8b	6.41	4.0	21.8	6.38	4.0	33.2	10.3	52	79
L2	C	10a	6.45	4.3	21.5	6.41	4.3	33.2	10.3	43	92
		10b	6.46	4.3	21.4	6.41	4.3	33.3	10.4	24	92
L2	C	12a	6.47	4.3	21.3	6.43	4.3	33.2	11.1	38	89
		12b	6.47	4.3	21.5	6.42	4.3	33.2	10.5	98	81
		average	6.44^{A}	4.1^{A}	21.6^{A}	6.41^{A}	4.1^{A}	33.2^{B}	10.9^{A}	47^{A}	80^{A}
		SD	0.02	0.2	0.5	0.01	0.2	0.1	0.4	20	12

continue

Cheesemaking trials			Cheesemaking parameters Curd processing (vat steps)									
Milk quality[1]	Climatic period[2]	trial nr	cooking temperature (°C)	cooking time (min)	rest under whey (min)	whey after cooking pH	whey after cooking titratable acidity	whey after cooking temperature (°C)	whey after curd extrac. pH	whey after curd extrac. acidity	whey after curd extrac. temp. (°C)	curd drainage time (min)
L1	C	1a	53.2	5.3	62	6.30	2.9	53.0	6.12	3.1	47.9	53
		1b	53.2	5.3	63	6.30	2.9	53.0	6.10	3.0	48.2	55
L1	W	3a	53.5	5.4	65	6.33	2.9	53.2	6.04	3.2	50.1	30
		3b	53.7	5.5	65	6.28	2.9	53.2	6.10	3.2	51.2	33
L1	W	5a	53.5	5.4	65	6.34	2.5	53.4	6.11	2.9	51.0	35
		5b	53.4	5.6	66	6.34	2.6	53.3	6.10	2.9	50.9	30
L1	W	7a	53.3	5.0	75	6.33	2.6	53.6	6.09	3.1	50.9	30
		7b	53.3	5.1	70	6.30	2.8	53.5	6.09	3.1	50.9	30
L1	C	9a	53.4	4.5	73	6.29	2.9	53.4	6.02	3.4	50.9	33
		9b	53.4	5.2	73	6.28	2.9	53.3	6.06	3.3	50.8	30
L1	C	11a	53.3	5.1	72	6.32	3.0	53.3	6.06	3.4	50.6	35
		11b	53.3	5.2	63	6.29	3.1	53.3	6.11	3.2	50.9	50
		average	53.4[A]	5.2[A]	68[A]	6.31[A]	2.8[A]	53.3[A]	6.13[A]	3.2[A]	50.4[A]	37[A]
		SD	0.1	0.3	5	0.02	0.2	0.2	0.15	0.2	1.1	10
L2	C	2a	53.4	5.2	60	6.30	2.8	53.0	6.11	3.0	50.5	40
		2b	53.3	5.0	65	6.30	2.8	53.0	6.09	3.0	49.0	32
L2	W	4a	53.4	5.4	69	6.30	2.8	53.0	6.10	3.0	50.4	22
		4b	53.7	4.6	71	6.31	2.8	53.4	6.15	3.1	51.0	22
L2	W	6a	53.8	5.5	70	6.31	2.6	53.7	6.10	2.9	51.0	33
		6b	53.8	5.1	65	6.31	2.7	55.1	6.15	2.8	52.6	40
L2	W	8a	54.0	5.0	75	6.29	2.7	53.8	6.04	3.1	51.2	43
		8b	53.5	5.1	77	6.30	2.9	53.3	6.01	3.2	51.0	35
L2	C	10a	53.6	5.0	70	6.31	2.9	53.5	6.05	3.2	50.4	45
		10b	53.4	5.2	62	6.31	2.9	53.4	6.08	3.1	50.9	40
L2	C	12a	53.3	5.0	73	6.32	2.6	53.3	6.14	2.9	51.2	25
		12b	53.5	5.1	71	6.32	2.6	53.5	6.12	3.0	51.0	20
		average	53.6[B]	5.1[A]	69[A]	6.31[A]	2.8[A]	53.5[A]	6.10[A]	3.0[B]	50.9[A]	33[A]
		SD	0.2	0.2	5	0.01	0.1	0.6	0.04	0.1	0.8	9

Cheesemaking trials			Cheesemaking parameters															
			Final cheese whey			Curd molding and salting					Curd composition at first turning							
Milk quality[1]	Climatic period[2]	trial nr	fat (%)	protein (%)	dry matter (%)	first turning (min)	second turning (min)	curd yield before salting (%)	salting time (d)	curd after salting yield (%)	pH	fat (%)	protein (%)	dry matter (%)	fat on DM, ratio	protein on DM, ratio	fat to protein, ratio
L1	C	1a	0.82	0.91	7.94	132	165	8.83	20.5	8.56	5.83	25.68	30.01	58.97	0.44	0.51	0.86
		1b	0.60	0.93	7.72	185	160	9.02	20.5	8.75	5.66	26.50	28.54	58.01	0.46	0.49	0.93
L1	W	3a	0.62	0.91	7.77	200	170	8.90	20.5	8.62	5.35	27.29	30.39	60.95	0.45	0.50	0.90
		3b	0.87	0.90	7.97	142	160	8.54	20.5	8.27	5.49	24.79	29.89	57.81	0.43	0.52	0.83
L1	W	5a	0.43	0.92	7.59	206	180	8.67	20.5	8.42	5.38	26.87	30.69	59.97	0.45	0.51	0.88
		5b	0.46	0.91	7.62	150	170	8.45	20.5	8.20	5.53	26.97	30.90	60.39	0.45	0.51	0.87
L1	W	7a	0.45	0.91	7.61	210	165	8.33	20.5	8.08	5.33	25.45	30.92	59.28	0.43	0.52	0.82
		7b	0.49	0.90	7.63	140	175	8.21	20.5	7.97	5.48	25.59	30.92	59.55	0.43	0.52	0.83
L1	C	9a	0.54	0.95	7.74	110	160	8.91	20.5	nd	5.27	27.47	30.77	62.15	0.44	0.50	0.89
		9b	0.44	0.95	7.68	167	165	9.09	20.5	nd	5.22	28.50	30.96	62.14	0.46	0.50	0.92
L1	C	11a	0.47	0.97	7.69	175	185	9.09	29.0	8.76	5.12	26.22	33.01	61.60	0.43	0.54	0.79
		11b	0.51	0.96	7.72	115	185	8.80	29.0	8.48	5.37	26.19	32.94	62.73	0.42	0.53	0.79
		average	0.56^{A}	0.93^{A}	7.72^{A}	161^{A}	170^{A}	8.74^{A}	21.9^{A}	8.41^{A}	5.42^{A}	26.46^{A}	30.83^{A}	60.29^{A}	0.44^{A}	0.51^{A}	0.86^{A}
		SD	0.15	0.02	0.12	35	9	0.30	3.3	0.27	0.19	1.03	1.22	1.65	0.01	0.01	0.05
L2	C	2a	0.68	0.93	7.79	145	165	8.60	20.5	8.25	5.47	24.03	31.66	58.56	0.41	0.54	0.76
		2b	0.66	0.93	7.76	203	165	8.50	20.5	8.15	5.40	23.54	30.92	57.30	0.41	0.54	0.76
L2	W	4a	0.40	0.95	7.56	195	180	8.55	20.5	8.28	5.29	25.15	30.98	58.69	0.43	0.53	0.81
		4b	0.45	1.00	7.95	138	175	8.67	20.5	8.40	5.37	24.99	30.90	59.19	0.42	0.52	0.81
L2	W	6a	0.43	0.95	7.49	220	160	8.31	20.5	8.05	5.24	25.39	31.97	59.74	0.42	0.54	0.79
		6b	0.42	0.95	7.48	165	150	8.40	20.5	8.14	5.62	25.20	31.25	59.58	0.42	0.52	0.81
L2	W	8a	0.38	0.97	7.50	132	185	8.33	20.5	8.08	5.15	23.07	33.29	60.34	0.38	0.55	0.69
		8b	0.39	0.96	7.50	205	185	8.21	20.5	7.96	5.09	22.64	32.66	58.94	0.38	0.55	0.69
L2	C	10a	0.44	1.03	7.64	180	180	8.89	28.0	8.56	5.19	24.51	33.27	60.37	0.41	0.55	0.74
		10b	0.46	1.03	7.64	135	180	9.09	28.0	8.75	5.24	24.14	32.73	59.85	0.40	0.55	0.74
L2	C	12a	0.44	1.01	7.64	140	180	9.06	21.5	8.78	5.21	24.07	32.35	58.85	0.41	0.55	0.74
		12b	0.40	1.02	7.60	140	180	8.95	21.5	8.64	5.19	24.43	32.48	59.20	0.41	0.55	0.75
		average	0.46^{A}	0.98^{B}	7.63^{A}	167^{A}	174^{A}	8.63^{A}	21.9^{A}	8.34^{A}	5.29^{A}	24.26^{B}	32.04^{B}	59.22^{A}	0.41^{B}	0.54^{B}	0.76^{B}
		SD	0.10	0.04	0.14	32	11	0.30	2.9	0.28	0.15	0.87	0.89	0.85	0.01	0.01	0.04

[1]milk from farms recognized for low (L1) or high (L2) content of butyric clostridia spores.

[2]climate of the period when the cheesemaking was carried out: warm (W), cool (C).

^{A,B} average values in column with different capital letters are different ($P < 0.05$)
