# Supplementary material for: Low-Level Clostridial Spores’ Milk to Limit the Onset of Late Blowing Defect in Lysozyme-Free, Grana-Type Cheese
Source: Foods. 2023 May 2;12(9):1880. doi: 10.3390/foods12091880 (PMC10177814; doi:10.3390/foods12091880)
Supplement: Supplementary file 1 [file foods-12-01880-s001.zip › Table S4.pdf]

Table S4. Chemical composition of ripened Grana-type cheese manufactured with L1 and L2 milk.

**Cheesemaking trials**			cheese ripening time (mo)	dry matter (%)	ash (%)	fat (%)	protein (%)	fat on DM (ratio	protein on DM (ratio)	fat to protein (ratio)	salt (%)			pH		
Milk quality[1]	Climatic period[2]	trial nr									overall sample	outer zone	inner zone	overall sample	outer zone	inner zone
L1	C	1	13	67.12	4.95	28.73	31.45	0.43	0.47	0.91	2.03	2.05	1.87	5.28	5.29	5.31
L1	W	3	11	67.50	4.80	29.74	31.10	0.44	0.46	0.96	1.80	1.90	1.45	5.39	5.39	5.42
L1	W	5	11	66.90	4.59	29.26	31.22	0.44	0.47	0.94	1.73	1.91	1.35	5.30	5.32	5.33
L1	W	7	10	66.71	4.38	28.13	32.39	0.42	0.49	0.87	1.34	1.62	1.08	5.46	5.47	5.52
L1	C	9	7	66.16	4.17	29.96	30.20	0.45	0.46	0.99	1.32	1.53	0.76	5.27	5.30	5.31
L1	C	11	7	66.41	4.63	28.54	31.51	0.43	0.47	0.91	1.75	2.14	1.06	5.36	5.36	5.33
		average	9.8^{A}	66.80^{A}	4.59^{A}	29.06^{A}	31.31^{A}	0.44^{A}	0.47^{A}	0.93^{A}	$1.66^{A,a}$	$1.86^{A,ab}$	$1.26^{A,b}$	$5.34^{A,a}$	$5.36^{A,a}$	$5.37^{A,a}$
		SD	2.4	0.48	0.28	0.72	0.71	0.01	0.01	0.04	0.28	0.24	0.38	0.07	0.07	0.08
L2	C	2	12	66.02	4.88	26.93	32.31	0.41	0.49	0.83	1.97	2.08	1.61	5.39	5.34	5.50
L2	W	4	11	67.22	4.59	28.60	32.30	0.43	0.48	0.89	1.64	1.88	1.11	5.47	5.42	5.87
L2	W	6	11	67.75	4.58	28.57	32.80	0.42	0.48	0.87	1.54	1.67	1.30	5.44	5.43	5.48
L2	W	8	10	65.57	4.66	26.56	32.52	0.41	0.50	0.82	1.69	1.93	1.08	5.61	5.41	5.84
L2	C	10	7	65.42	4.91	26.72	32.11	0.41	0.49	0.83	2.10	2.17	0.97	5.42	5.31	5.80
L2	C	12	7	64.09	4.68	25.69	31.87	0.40	0.50	0.81	1.72	1.99	1.21	5.30	5.29	5.34
		average	9.8^{A}	66.01^{A}	4.72^{A}	27.18^{B}	32.32^{B}	0.41^{B}	0.49^{B}	0.84^{B}	$1.78^{A,a}$	$1.95^{A,a}$	$1.21^{A,b}$	$5.44^{A,a}$	$5.37^{A,ab}$	$5.64^{B,b}$
		SD	2.3	1.32	0.15	1.17	0.32	0.01	0.01	0.03	0.21	0.18	0.22	0.10	0.06	0.23

Cheesemaking trials			Organic acids (g on kg DM)													
			lactic			butyric			propionic			acetic			succinic	pyroglutamic
Milk quality[1]	Climatic period[2]	trial nr	overall sample	outer zone	inner zone	overall sample	outer zone	inner zone	overall sample	outer zone	inner zone	overall sample	outer zone	inner zone	overall sample	overall sample
L1	C	1	26.73	25.76	25.97	0.000	0.000	0.000	0.000	0.000	0.000	1.862	1.654	2.011	0.998	4.991
L1	W	3	24.49	23.13	23.51	0.000	0.000	0.385	0.000	0.000	0.000	2.104	1.941	2.133	0.978	4.296
L1	W	5	24.34	24.49	23.57	0.000	0.000	0.000	0.000	0.000	0.000	1.958	1.854	1.869	0.972	3.991
L1	W	7	24.16	24.61	21.80	0.000	0.000	1.124	0.000	0.000	0.000	2.189	2.174	1.934	0.869	3.747
L1	C	9	25.45	23.75	24.90	0.000	0.000	0.000	0.000	0.000	0.000	1.255	1.224	0.982	0.892	2.962
L1	C	11	23.37	21.62	24.21	0.000	0.000	0.000	0.000	0.000	0.000	1.717	1.671	1.476	0.873	2.545
		average	24.76^{A,a}	23.89^{A,a}	23.99^{A,a}	0.000^{A,a}	0.000^{A,a}	0.252^{A,a}	0.000^{A,a}	0.000^{A,a}	0.000^{A,a}	1.847^{A,a}	1.753^{A,a}	1.734^{A,a}	0.930^{A}	3.756^{A}
		SD	1.17	1.42	1.41	0.000	0.000	0.454	0.000	0.000	0.000	0.336	0.322	0.430	0.058	0.891
L2	C	2	25.70	25.58	21.73	0.000	0.000	2.302	0.000	0.000	0.000	2.014	1.787	1.742	1.045	4.529
L2	W	4	21.66	22.18	10.49	1.190	0.000	5.921	0.000	0.000	1.041	1.904	1.979	1.205	0.937	4.017
L2	W	6	23.64	24.06	22.15	0.000	0.000	0.443	0.000	0.000	0.000	2.125	1.963	2.111	0.871	3.778
L2	W	8	23.17	24.36	13.33	1.586	0.000	6.024	0.244	0.000	1.190	2.105	1.983	1.769	0.808	4.255
L2	C	10	21.22	23.07	7.87	1.727	0.000	7.352	0.290	0.000	1.513	1.590	1.697	0.810	0.764	2.583
L2	C	12	25.74	25.09	24.53	0.328	0.000	1.716	0.000	0.000	0.218	2.013	1.888	1.763	0.858	2.684
		average	23.52^{A,a}	24.06^{A,a}	16.68^{B,b}	0.805^{A,a}	0.000^{A,a}	3.960^{B,b}	0.089^{A,a}	0.000^{A,a}	0.660^{A,a}	1.959^{A,a}	1.883^{A,a}	1.567^{A,a}	0.881^{A}	3.641^{A}
		SD	1.93	1.26	6.99	0.792	0.000	2.820	0.139	0.000	0.666	0.197	0.118	0.471	0.100	0.820

[1]milk from farms recognized for low (L1) or high (L2) content of butyric clostridia spores.
[2]climate of the period when the cheesemaking was carried out: warm (W), cool (C).

^{A,B} average values in column with different capital letters are different ($P < 0.05$)
^{a,b} for parameters measured in different layers of each cheese sample, average values in row with different lowercase letters are different ($P < 0.05$)
