# Supplementary figures and images for: Low-Level Clostridial Spores’ Milk to Limit the Onset of Late Blowing Defect in Lysozyme-Free, Grana-Type Cheese
Source: Foods. 2023 May 2;12(9):1880. doi: 10.3390/foods12091880 (PMC10177814; doi:10.3390/foods12091880)

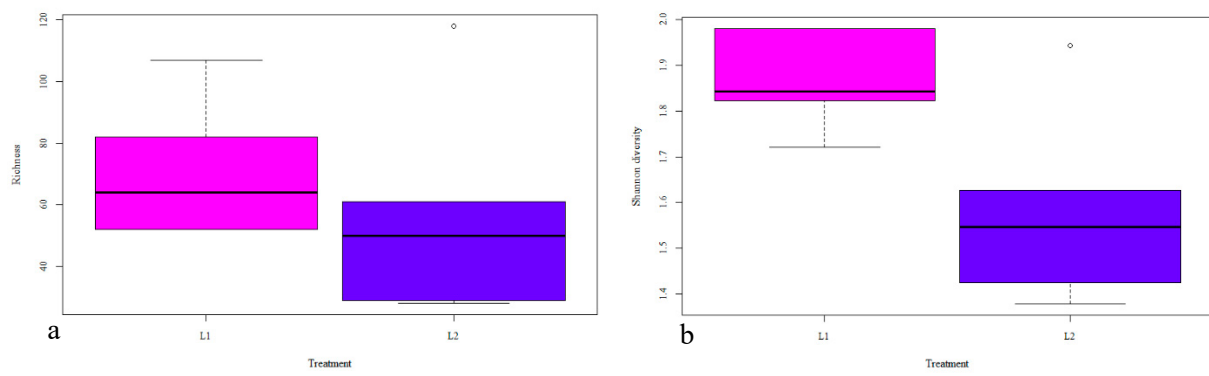

Figure S2. Richness (a) and Shannon diversity (b) of L1 and L2 Grana-like cheese samples.

Supplement: Supplementary file 1 [file foods-12-01880-s001.zip › Figure S2.pdf]
